# Organic Heterostructures with Dendrimer Based Mixed Layer for Electronic Applications

**DOI:** 10.3390/molecules29174155

**Published:** 2024-09-01

**Authors:** Oana Rasoga, Anne Lutgarde Djoumessi Yonkeu, Carmen Breazu, Marcela Socol, Nicoleta Preda, Florin Stanculescu, Anca Stanculescu, Emmanuel Iwuoha

**Affiliations:** 1 National Institute of Materials Physics, 405A Atomistilor Street, P.O. Box MG-7, 077125 Magurele, Romania; oana.rasoga@infim.ro (O.R.); carmen.breazu@infim.ro (C.B.); marcela.socol@infim.ro (M.S.); nicol@infim.ro (N.P.); 2 SensoLab, Department of Chemistry, University of Western Cape, Robert Sobukwe Road, Bellville, Cape Town 7535, South Africa; 3116018@myuwc.ac.za (A.L.D.Y.); eiwuoha@uwc.ac.za (E.I.); 3 Faculty of Physics, University of Bucharest, 405 Atomistilor Street, P.O. Box MG-11, 077125 Magurele, Romania; fstanculescu@fpce1.fizica.unibuc.ro

**Keywords:** organic heterostructure, dendrimers, non-fullerene acceptor, bulk heterojunction

## Abstract

Recently, much research has focused on the search for new mixed donor–acceptor layers for applications in organic electronics. Organic heterostructures with layers based on the generation 1 poly(propylene thiophenoimine) (G1PPT) dendrimer, N,N′-diisopropylnaphthalene diimide (MNDI), and a combination of the two were prepared and their electrical properties were investigated. Single layers of G1PPT and MNDI and a mixed layer (G1PPT:MNDI) were obtained via spin coating on quartz glass, silicon, and glass/ITO substrates, using chloroform as a solvent. The absorption mechanism was investigated, the degree of disorder was estimated, and the emission properties of the layers were highlighted using spectroscopic methods (UV–Vis transmission and photoluminescence). The effects of the concentration and surface topographical particularities on the properties of the layers were analyzed via atomic force microscopy. All of the heterostructures realized with ITO and Au electrodes showed good conduction, with currents of the order of mA. Additionally, the heterostructure with a mixed layer exhibited asymmetry in the current–voltage curve between forward and reverse polarization in the lower range of the applied voltages, which was more significant at increased concentrations and could be correlated with rectifier diode behavior. Consequently, the mixed-layer generation 1 poly(propylene thiophenoimine) dendrimer with N,N′-diisopropylnaphthalene diimide can be considered promising for electronic applications.

## 1. Introduction

Due to their specific properties, dendrimers are a class of molecules that represent interesting candidates for light-absorbing, charge-transporting, and electron-donating materials, with a wide range of potential applications, from sensing and organic optoelectronics to drug delivery systems and (bio)medicine [1,2,3,4,5,6,7,8]. For example, in the healthcare field, dendrimers are promising not only in multifunctional drug delivery [9] but also in the development of new forms of personalized medicine [10]. Recently, the medical applications of dendrimers have been extended to COVID-19 treatment through the study of dendrimer–peptide conjugates, which block the interaction of the spike protein of the virus with the human receptor [11]. Dendrimers are interesting in the development of photoinitiating systems for the activation of polymerization, enabling the use of eco-friendly conditions and avoiding toxic compounds [12]. Because some π-conjugated dendrimers are characterized by high absorption in a wide region of the solar spectrum [13], they could be promising materials for organic solar cells [14,15,16]. Special attention has been paid to the synthesis of dendrimers with emission in different regions of the spectrum, i.e., red, green, or blue, for use in the realization of simple OLED structures, showing good efficiency [17,18,19,20,21]. The effect of the dendrimer surface groups on the transport and photophysical properties of the emissive layers composed of blends (mixtures) based on dendrimers has also been investigated [22]. Recently, research has been focused on hyperfluorescent OLEDs, which are based on fluorescent dendrimers, showing the phenomenon of thermally activated delayed fluorescence (TADF) in an assistant host [23].

The specific configuration of dendrimers determined by repeating subunits starting from a central core with several branching interior layers and specific groups at their surfaces provides them with unique properties. This configuration allows the size and molecular weight of the self-assembling system to be changed, making it suitable for a target function [24]. Thus, dendrimers are suitable for compact nanometer-scale devices because of this well-defined pattern, as well as their controlled shape and size, which is of the order of a few nanometers [25]. Due to their branched macromolecular structures and the extended conjugation of the linear arms (dendrons), dendrimers provide efficient intermolecular overlapping, favoring high three-dimensional (3D) electrical conductivity [26,27,28] and overcoming the main limitations of organic compounds related to anisotropy and charge carrier transport.

Studies have revealed that high-generation dendrimers are not absolutely necessary for some practical applications [13]. The structural and synthesis particularities of dendrimers favor the introduction of the desired functionalities in low-generation dendrimers [13]. Thus, they are expected to be more suitable for optoelectronic applications, as the lower generation of dendrimers contains branched macromolecules with good electronic and optoelectronic properties in their structures [13].

Thus, the proposed donor is a low-generation dendrimer obtained through the functionalization of the generation 1 poly(propyleneimine) tetraamine with thiophene units. Both the core (propyleneimine) and the dendrons (thiophene groups) exhibit certain optical properties; the dendrons exhibit charge transport and the surface groups display processing properties [28]. The adequate choice of the surface groups favors good solubility and easy solution processing [28]. In general, dendrimers are characterized by a low position on the energy scale of the highest occupied molecular orbital (HOMO) level, and the charge carrier transport is influenced by the trapping mechanism. If the HOMO level is lower than 6 eV on the energy scale, hole transport is limited by hole trapping at an energy level that is likely determined by water clusters [29]. A balance must be considered between the positive effect of the 3D conduction associated with the dendrimer structure and the charge carrier transport limitation, i.e., hole-limited transport, which is caused by the low HOMO level. Cyclic voltammetry measurements with a Au working electrode have revealed the values of $E_{ox\;}^{onset}$ and $E_{red}^{onset}$ for G1PPT [30]. These values have been corrected for the Ag/AgCl reference electrode [31,32] and are introduced in the following formulas [33,34]:

(1) $$E_{LUMO}=4.34-\left(E_{red}^{onset}\right)\;\mathrm{eV},$$

(2) $$E_{HOMO}=4.34+\left(E_{ox}^{onset}\right)\;\mathrm{eV},$$

resulting in the following values for the HOMO and the lowest unoccupied molecular orbital (LUMO): *E*_*HOMO*,*G1PPT*_ = 6.45 eV and *E*_*LUMO*,*G1PPT*_ = 2.99 eV.

The good 3D transport anticipated for the donor consisting of the proposed generation 1 dendrimer based on propylene thiophenoimine overcomes the limitations associated with having a HOMO level lower than 6 eV on the energy scale. Deposition from a solution is favored by the improvement in the solubility determined by the thiophene surface groups.

Fullerene and fullerene derivatives are the most used acceptors in mixtures with donors to generate bulk heterojunction active layers. Although fullerene-based acceptors show good, trap-free electron transport, they also have some drawbacks, among which are their low optical absorption and low solubility, affecting the morphology. Recently, special attention has been paid to the identification and investigation of non-fullerene acceptors (NFAs). These compounds show great potential to replace fullerene materials and overcome their limitations. Additionally, the structural diversity of these compounds assures the fine tuning of the energy levels’ positions.

The proposed acceptor is a small-molecule, solution-processable naphthalene diimide derivative [35,36] that combines high absorption and solubility with tunable material properties, e.g., the LUMO level, which is associated with the electron-deficient nature of the compound and its very high π-acidity [37]. N,N-dialkylnaphthalene diimides represent a class of six-member aromatic compounds with strong potential for the formation of electron-accepting semiconductor materials. The alkyl chains, due to their conformational flexibility, can result in increased solubility [38] without preventing the crystalline packing of the π-conjugated cores [39]. The LUMO level must be lower than 3.6 eV because organic semiconductors are generally characterized by an electron trapping level of ~3.6 eV [40]. This level, which is associated with the presence of hydrated oxygen complexes, is assumed as a universal trap center, not only in polymers but in all organic semiconductors [41,42]. The electrons recombine at this level, and compounds with a LUMO level that is higher on the energy scale will be characterized by electron trap-limited transport [29]. The proposed N,N′-diisopropylnaphthalene diimide satisfies this condition; additionally, it shows good solubility due to the chemical modification introduced by the use of an aliphatic substituent, preventing aggregation [43]. Different LUMO values for MNDI, such as 4.08 eV [44] or 3.66 eV [45], are mentioned in the literature. Furthermore, the compound is resistant to oxidation [44] because it is characterized by a high HOMO level of 6.36 eV [45]. 

The single layers based on a low-generation dendrimer of propylene thiophenoimine (G1PPT) and diisopropylnaphthalene diimide (MNDI) and mixed layers G1PPT:MNDI were prepared from solutions of different concentrations via spin coating, and their optical properties were investigated, focusing on the influence of the layers’ morphology. The electrical properties of the heterostructures with these single and mixed layers between ITO and Au electrodes were analyzed, revealing good conduction and ohmic or rectifying behavior, which are promising features for potential electronic applications.

## 2. Results

### 2.1. Optical Characterization

#### 2.1.1. Transmission of Single and Mixed Layers

**Figure 1:**
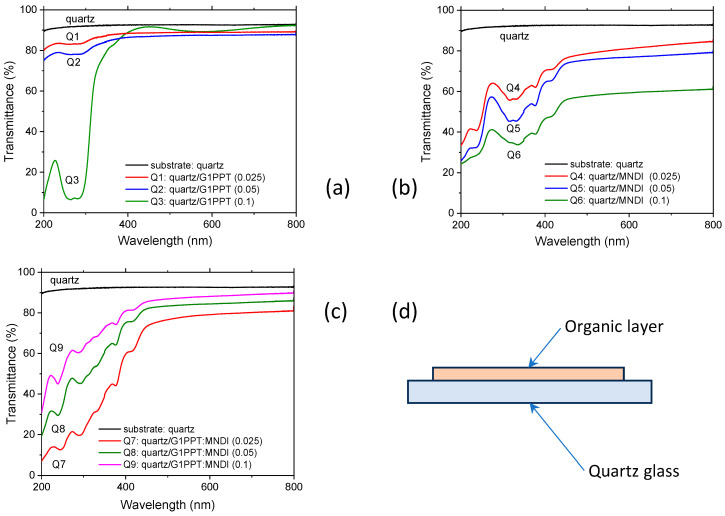
UV–Vis transmission spectra of layers deposited on quartz from solutions with different concentrations: (**a**) G1PPT [Q1: 0.025 g/2 mL, Q2: 0.05 g/2 mL, Q3: 0.1 g/2 mL]; (**b**) MNDI [Q4: 0.025 g/2 mL, Q5: 0.05 g/2 mL, Q6: 0.1 g/2 mL]; (**c**) G1PPT:MNDI mixed layer [Q7: 0.025 g/2 mL: 0.025 g/2 mL, Q8: 0.05 g/2 mL: 0.05 g/2 mL, Q9: 0.1 g/2 mL: 0.1 g/2 mL]. (**d**) Schematic representation of the investigated samples. The codes of the samples are presented in Table 1.

The transmission of the G1PPT layers on quartz glass (Figure 1a) showed a UV absorption band between 250 and 300 nm, with two peaks, as indicated in the literature [46,47]. This band is well structured in the thicker layer deposited from a higher-concentration solution (0.1 g/2 mL). Independent of the concentration, the MNDI layers show a large, structured absorption band situated between 250 and 500 nm, with many local maxima at 236, 317, 336, 377, and 417 nm (Figure 1b). The shape of the absorption edge for the mixed layers of G1PPT:MNDI was determined through the superposition of the behavior of the two components, G1PPT and MNDI (Figure 1c), and the mixed layers showed good absorption at wavelengths lower than 500 nm.

**Figure 2:**
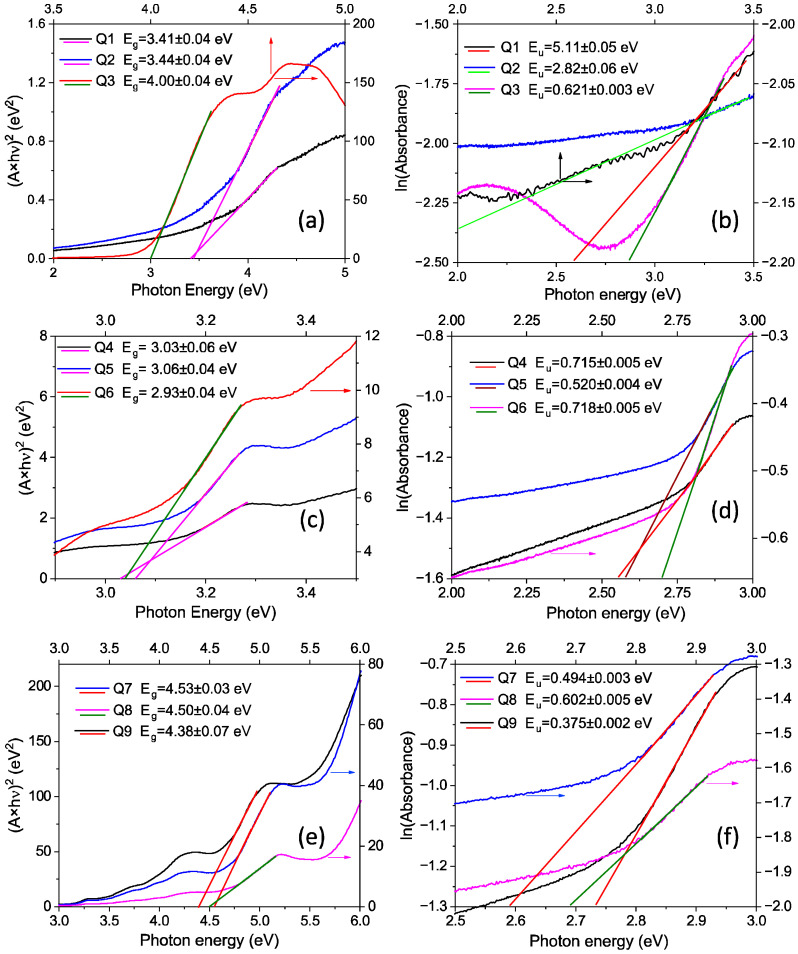
Tauc and Urbach plots for G1PPT (**a**,**b**), MNDI (**c**,**d**), and G1PPT:MNDI (**e**,**f**) at different concentrations (Q1, Q4, Q7: 0.025 g/2 mL; Q2, Q5, Q8: 0.05 g/2 mL; and Q3, Q6, Q9: 0.1 g/2 mL). All layers were deposited on quartz glass, which is characterized by a wide optical band gap; therefore, this did not affect the results in the investigated range of photon energies. The codes of the samples are presented in Table 1.

Some details regarding the intrinsic properties of the compounds and the quality of the layers were obtained from the UV–Vis spectra when they were deposited via spin coating on a quartz glass substrate characterized by a very large transparency domain from UV to NIR. The optical band gap and degree of disorder were evaluated by applying the Tauc [48] and Urbach [49] plots, respectively, to the layers prepared from solutions with the selected concentrations (0.025 g/2 mL, 0.5 g/2 mL, and 0.1 g/2 mL) (Table 1). The linear region of the plot (absorbance × E)^1/r^ as a function of E, where E = photon energy and r is a coefficient reflecting the correlation with the light absorption mechanism, was interpolated in the high-photon energy range >3.0 eV (Figure 2a,c,e). All possible absorption mechanisms, encompassing direct and indirect allowed and forbidden transitions, were tested. The transition characterized by r = 1/2 (direct, allowed transition) allowed the best linear fitting of the data for the samples with G1PPT, MNDI, and G1PPT–MNDI layers. The plot of ln(absorbance) as a function of E for a photon energy of <3.0 eV (Figure 2b,d,f) gives the value of the Urbach energy, E_u_. The Urbach energy is the inverse of the slope of the interpolation line drawn in the linear region of the plot and is correlated with the degree of static (structural and morphological) disorder.

G1PPT showed a band gap, E_g,G1PPT_, that increased from 3.4 eV to 4 eV with the increasing concentration and an E_u_ energy level that also increased with the increasing concentration (Figure 2a,b). The layers of MNDI revealed a fundamental absorption edge at around 3 eV (Figure 2c), the position of which was not significantly affected by the concentration. The highest E_u_ value was found at a concentration of 0.1 g/2 mL (Figure 2d). The absorption threshold of the mixed layers was situated at a photon energy higher than 4 eV (Figure 2e), and the lowest E_u_ was obtained at a concentration of 0.1 g/2 mL (Figure 2f).

#### 2.1.2. Photoluminescence Spectra of Single and Mixed Layers

**Figure 3:**
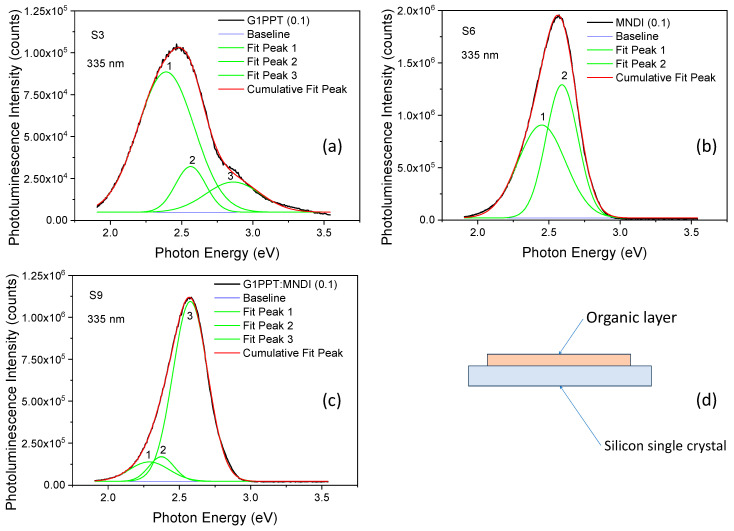
Typical deconvoluted photoluminescence spectra of samples prepared on Si substrate from solutions with concentrations of 0.1 g/2 mL at excitation with λ = 335 nm: (**a**) G1PPT; (**b**) MNDI; (**c**) G1PPT:MNDI mixed layer. (**d**) Schematic representation of the investigated samples. The codes of the samples are presented in Table 2.

All layers showed photoluminescence (PL) emission with a peak situated at an excitation wavelength of around 480–500 nm in the UV region; λ_exc_ = 335 nm (3.70 eV) (Figure 3). The Si substrate did not contribute to the emission spectra. Radiation at 335 nm was located in the excitation band for both the donor (315–440 nm [46]) and acceptor (300–400 nm [30]). The asymmetry of the emission spectra suggested the presence of hidden peaks, and deconvolution was used to process the PL data of the deposited layers to highlight these peaks.

Independent of the concentration, the layers of G1PPT showed three emission peaks (Figure 3a), the layers of MNDI showed two emission peaks (Figure 3b), and the mixed layers of G1PPT:MNDI showed three emission peaks (Figure 3c). The results of the deconvolution of the emission peaks at excitation at 335 nm are presented in Table 2. 

### 2.2. Structural Properties of Single and Mixed Layers

**Figure 4:**
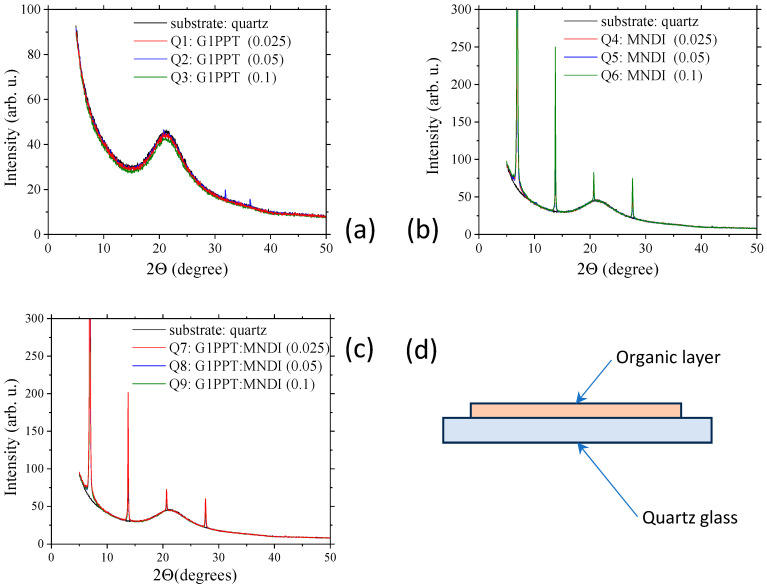
X-ray diffractograms of layers deposited on quartz from solutions with different concentrations: (**a**) G1PPT [Q1—0.025 g/2 mL, Q2—0.05 g/2 mL, Q3—0.1 g/2 mL]; (**b**) MNDI [Q4—0.025 g/2 mL, Q5—0.05 g/2 mL, Q6—0.1 g/2 mL]; (**c**) G1PPT:MNDI mixed layer [Q7—0.025 g/2 mL:0.025 g/2 mL, Q8—0.05 g/2 mL:0.05 g/2 mL, Q9—0.1 g/2 mL:0.1 g/2 mL]. (**d**) Schematic representation of the investigated samples. The codes of the samples are presented in Table 1.

The presence of a disorder or a certain degree of ordering in the layers was revealed by the XRD measurements (Figure 4), with the structural characteristics affecting their properties. On the X-ray diffractograms of the G1PPT films deposited via spin coating on quartz glass, we did not identify any peaks, which indicated that the layer was disordered. We identified only a wide peak centered at 21°, associated with the X-ray diffraction of the quartz substrate (Figure 4a). By contrast, the X-ray diffractograms of the MNDI layer deposited via spin coating on quartz glass using the “mother solutions” of different concentrations revealed strong narrow peaks positioned at ~7°, 13.8°, 20.7°, and 27.6° (Figure 4b). These peaks associated with MNDI were also revealed in the diffractograms of the mixed layer (Figure 4c).

### 2.3. Morphological Properties of Single and Mixed Layers

**Figure 5:**
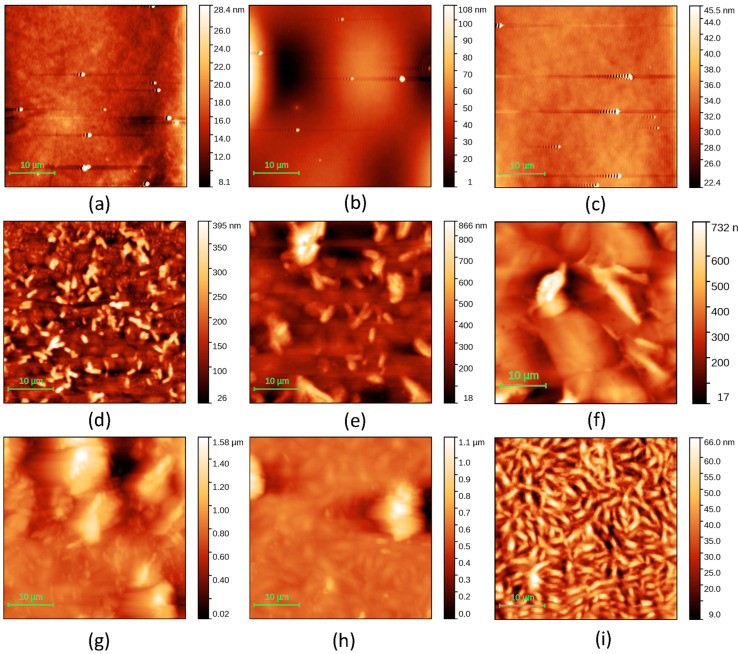
Two-dimensional AFM images of layers deposited on glass/ITO from solutions with different concentrations: G1PPT [(**a**) P1—0.025 g/2 mL, (**b**) P2—0.05 g/2 mL, (**c**) P3—0.1 g/2 mL]; MNDI [(**d**) P4—0.025 g/2 mL, (**e**) P5—0.05 g/2 mL, (**f**) P6—0.1 g/2 mL]; G1PPT:MNDI [(**g**) P7—0.025 g/2 mL:0.025 g/2 mL, (**h**) P8—0.05 g/2 mL:0.05 g/2 mL, (**i**) P9—0.1 g/2 mL:0.1 g/2 mL]. The codes of the samples are presented in Table 3.

The surface topography of the layers was observed through AFM measurements. The surface roughness affects the contact between the layers and electrodes, and thus, the charge carrier injection and collection. The dendrimer layers obtained via spin coating on glass/ITO were very smooth (Figure 5a–c; Table 3) compared to the layers of the naphthalene diimide derivative deposited via spin coating on the same type of substrate (Figure 5d–f; Table 3).

**Figure 6:**
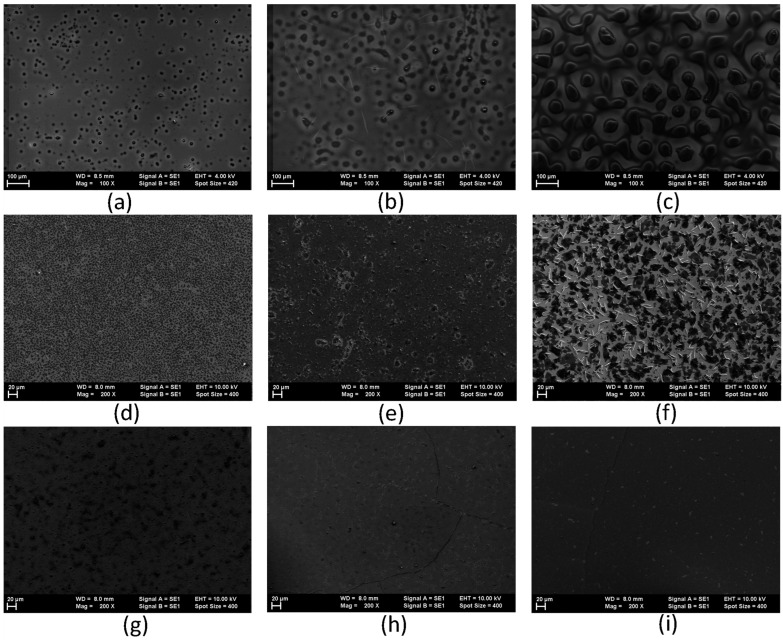
SEM images of a single layer of G1PPT (**a**–**c**), a single layer of MNDI (**d**–**f**), and a mixed layer of G1PPT:MNDI (**g**–**i**) on Si for concentrations of 0.025 g/2 mL, 0.05 g/2 mL, and 0.1 g/2 mL.

The SEM images of the G1PPT layers deposited on Si revealed the presence of clusters of G1PPT molecules (Figure 6a–c), whose dimensions and density increased with the concentration. The MNDI layers deposited on Si were characterized by a platelet-like morphology overlapped by stick-like grains (Figure 6d–f). The mixed layers of G1PPT:MNDI showed a morphology characterized by aggregates that were relatively homogeneously spread throughout the layer (Figure 6g–i).

**Figure 7:**
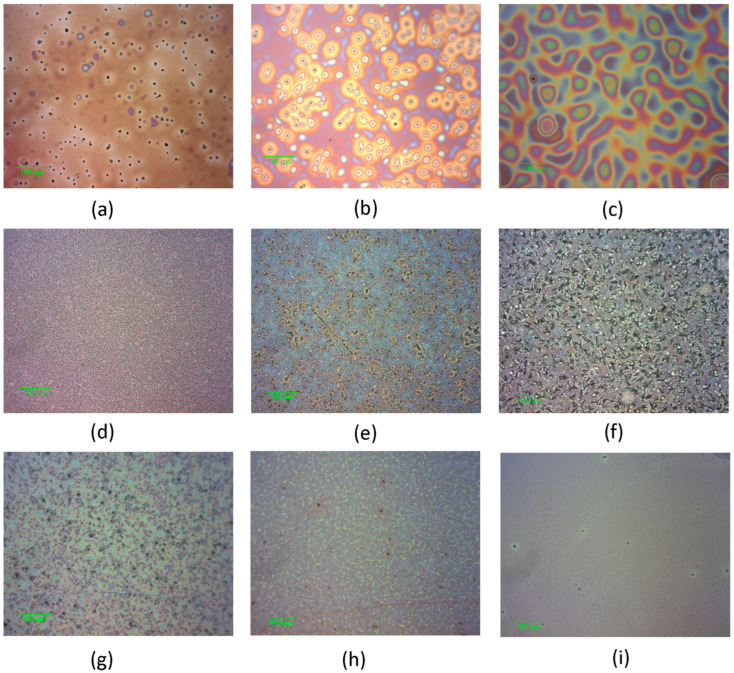
Optical images of layers deposited on glass/ITO from solutions with different concentrations: G1PPT [(**a**) P1—0.025 g/2 mL; (**b**): P2—0.05 g/2 mL; (**c**) P3—0.1 g/2 mL]; MNDI [(**d**) P4—0.025 g/2 mL; (**e**) P5—0.05 g/2 mL; (**f**) P6—0.1 g/2 mL]; G1PPT:MNDI [(**g**) P7—0.025 g/2 mL:0.025 g/2 mL; (**h**) P8—0.05 g/2 mL:0.05 g/2 mL; (**i**) P9—0.1 g/2 mL:0.1 g/2 mL]. The codes of the samples are presented in Table 3.

The optical images (Figure 7) highlight the morphological particularities of the layers deposited on glass/ITO, emphasizing the presence of aggregates in the layers of MNDI (Figure 7d–f) and mixed layers of G1PPT:MNDI (Figure 7g–i).

## 3. Electrical Characterization

The electrical measurements were conducted in a vertical configuration between the two electrodes (ITO and Au), with the area limited by the area of the Au electrode. The gold contact was circular and situated in the center of the deposited layer, with an area of 0.28 cm^2^, which was much smaller than the area of the deposited layer of 3 cm^2^. Thus, there was no possibility for the Au electrode to be in contact with the ITO electrode, generating a short circuit. 

**Figure 8:**
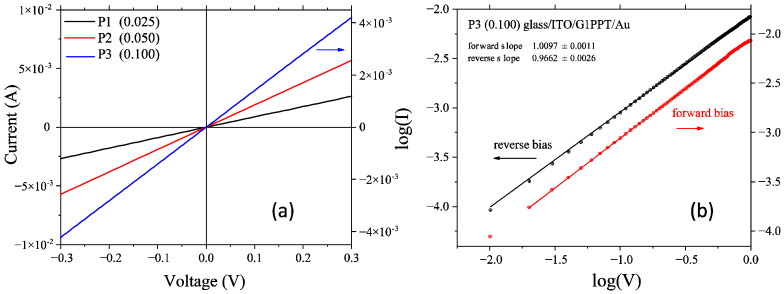
Electrical properties of the glass/ITO/G1PPT/Au heterostructure. (**a**) I–V characteristics for different concentrations of the active layer; (**b**) log(I)–log(V) representation at forward (ITO−, Au+) and reverse (ITO+, Au−) polarization for a concentration of 0.1 g/2 mL.

Charge transport in organic devices is influenced by charge injection from the contacts and the charge carriers’ movement in the bulk of the organic layer, represented by π-conjugated materials [50,51]. The almost linear shape (Figure 8a) of the I–V curve for voltages < 0.3 V is correlated with the ohmic contact behavior of the ITO/G1PPT/Au heterostructure when the layer of G1PPT is deposited from the low-concentration solution (0.025 g/2 mL). When the dendrimer layer is deposited from solutions with concentrations of 0.05 g/2 mL and 0.1 g/2 mL, this behavior does not change significantly for voltages < 0.3 V (Figure 8a).

**Figure 9:**
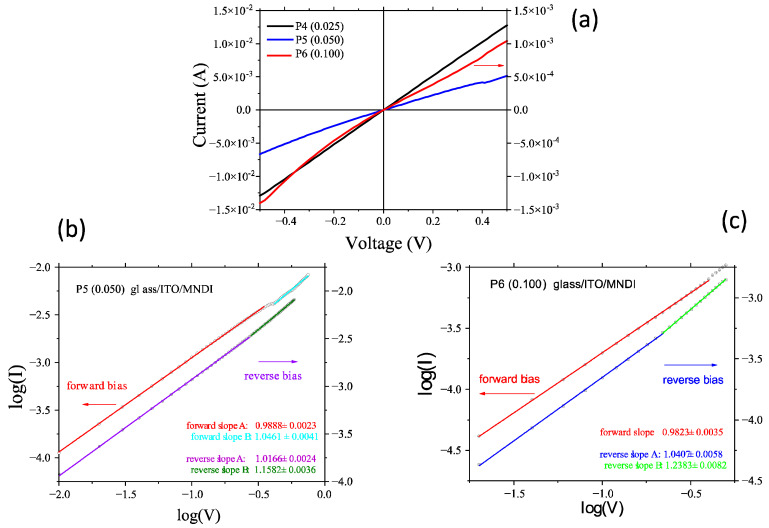
Electrical properties of the glass/ITO/MNDI/Au heterostructure. (**a**) I–V characteristics for different concentrations of the active layer; (**b**) log(I)–log(V) representation at forward (ITO−, Au+) and reverse (ITO+, Au−) polarization for a concentration of 0.05 g/2 mL; (**c**) log(I)–log(V) representation at forward and reverse polarization for a concentration of 0.1 g/2 mL.

The I–V characteristic of the ITO/MNDI/Au heterostructure at a low concentration (0.025 g/2 mL) is practically linear and symmetric (Figure 9a). With the increase in the concentration (0.05 g/2 mL), the I–V characteristic becomes slightly non-linear and asymmetric (Figure 9a). This behavior is strengthened at higher concentrations (0.1 g/2 mL). The log(I)–log(V) representations provide confirmation of the behavior of the heterostructures at direct and reverse polarization (Figure 9b,c).

The heterostructure with a mixed layer and a concentration of 0.1 g/2 mL showed a current of 6 × 10^−3^ A for an applied voltage of 0.5 V. This indicates good conduction if it is considered that, when traveling to the electrode, some charge carriers can be lost due to recombination at the grain boundaries shown in the SEM images (Figure 6g–i).

Both the ITO/organic mixed layer and organic mixed layer/Au are Schottky diodes. Using the Schottky equation, we calculated the ideality factor, n, of the diode with the G1PPT:MNDI mixed layer (Table 4). The ideality factor is given by the formula $n=\frac{q}{kTp}$, where q = electron charge, T = absolute temperature, k = the Boltzmann constant, and $p=\frac{d\left(lnI\right)}{dV}$, which is the slope of the linear portion of the ln(I) = f(V) curve [52,53].

## 4. Discussion

The absorption band of G1PPT, situated between 300 and 500 nm (Figure 1a), is well structured in the thicker layer deposited from a slightly more viscous solution (0.1 g/2 mL); this is due to the stronger intermolecular interactions determined by the more densely packed arrangement of the molecules in the layer. The absorption band is attenuated when the concentration of G1PPT in Ch decreases because the molecules are located at a greater distance from each other and the interactions are weaker in the layers obtained from more dilute solutions (Figure 1a). This band is assigned to the absorption of the thiophene-functionalized dendrimer [46,47,54]. The peak positioned at ~260 nm is characteristic of all thiophenes and is due to the π–π* transition in the thiophene ring [54,55,56]. The transmittance loss is caused by the absorption and scattering of light in the thickness of the dendrimer layer. The absorption band situated between 270 and 290 nm, with a peak at ~280 nm, could be correlated with the low-lying electronic n–π* transition of the C-S-C chromophore and associated with the excitation of lone pairs of electrons on the heteroatoms of S. This transition is forbidden under symmetry considerations; the corresponding band is weak in intensity and could be partially covered by the higher-intensity band attributed to the π–π* electronic transition.

In the spectrum of MNDI (Figure 1b), the peak situated at ~230 nm can be associated with the n–π* transition of the carbonyl group [57], the peak situated at ~315 nm with naphthalene [58], and the peaks situated at ~340 nm and ~380 nm with naphthalene–imide moiety interactions [43,59]. The peak located between 400 nm and 450 nm, namely, at ~410 nm, may be due to a localized π–π* transition involving the MNDI units. These peaks can be shifted because of the stronger intermolecular interaction and packing in the solid state, associated with the degree of the molecules’ organization and the specific type of aggregation in the layer [60].

The weak absorption bands situated between 250 and 500 nm in the spectrum of the G1PPT:MNDI mixed layer (Figure 1c) could be associated with a fundamental absorption edge in steps characterizing the naphthalene diimide derivative (Figure 1b). The behavior of MNDI is dominant because its transmission spectrum shows many absorption bands over 300 nm, while the transmission spectrum of G1PPT does not show any characteristic well-defined absorption bands over 300 nm (Figure 1a). The only exception for G1PPT is the absorption band situated at wavelengths lower than 300 nm, which is well defined in the spectrum of the layer deposited from the highest-concentration solution, characterized by stronger absorption. 

The band gap values of G1PPT and MNDI are similar to those previously presented of 3.38 eV and 3.16 eV [47] for the low-concentration solutions (0.025 and 0.05 g/2 mL). Differences appeared for both compounds at a higher concentration (0.1 mg/2 mL): 4 eV for G1PPT and 2.93 eV for MNDI. The widest forbidden band, >4 eV, was revealed by the mixed layers for all concentrations. In the mixed layer, the band gap was wider compared to the individual components, namely, G1PPT (Figure 2a) and MNDI (Figure 2c), and it slightly decreased with an increase in concentration (Figure 2e). This can be explained by the certain degree of order generated in the layer by mixing, limiting the introduction of the trap levels.

While the band gap of the G1PPT layer increased with increasing concentration, the band gap of the G1PPT:MNDI layer slightly decreased.

In the layer of G1PPT prepared from solutions with low concentrations (0.025 g/2 mL and 0.05 g/2 mL), the molecules were likely to be very far apart and not tightly packed. Therefore, the results obtained when processing the experimental UV–Vis data indicated a high value for the Urbach energy, which is correlated with a significant degree of disorder (Figure 2b). At a higher concentration, E_u_ decreased, indicating a decreased disorder and increased quality of the dendrimer layer. This can be correlated with the increase in the band gap. The layer of MNDI showed, at low concentrations (0.25 g/2 mL and 0.5 g/2 mL), a lower degree of disorder (Figure 2d) compared to the G1PPT layer at the same concentration. In contrast to the G1PPT layer, the highest Urbach energy and disorder were obtained for the MNDI layer at the highest concentration of 1 g/mL (Table 1) because the deposition process favored the formation of large aggregates and clusters of grains and the light was scattered across the grain boundaries. This can be correlated with a decrease in the band gap. The lowest disorder in the mixed layer, E_u_ = 0.375, was obtained at the concentration of 0.1 g/2 mL (Figure 2f). High concentrations favor the agglomeration of molecules during deposition and the formation of large aggregates, introducing a reduced number of boundaries for radiation scattering. 

The lowest E_u_ value, correlated with a reduced degree of disorder, was obtained at a concentration of 0.1 g/2 mL for the G1PPT and G1PPT:MNDI layers. For the MNDI layers, at a concentration of 0.1 g/2 mL, the highest E_u_ was obtained.

At an excitation of 335 nm, the G1PPT layer deposited from the solution with a concentration of 0.025 g/2 mL showed three peaks, situated at 2.41 eV, 2.57 eV, and 2.88 eV, with a full width at half maximum (FWHM) of 0.47 eV, 0.25 eV, and 0.43 eV, respectively (Table 2). The MNDI layer with a concentration of 0.025 g/2 mL showed two peaks, situated at 2.42 eV and 2.58 eV, with an FWHM of 0.44 eV and 0.26 eV, respectively (Table 2). The shape and intensity of the emission of MNDI were probably due to excimer-like emission [61], which are present in the solid state. The emission peaks’ intensity increased with concentration for G1PPT and MNDI because the number of excited molecules that could relax to the ground state increased. The emission spectra of the mixed layer showed three peaks (Figure 3c). For a concentration of 0.025 g/2 mL, these peaks were situated at 2.30 eV, 2.37 eV, and 2.57 eV with an FWHM of 0.33 eV, 0.21 eV, and 0.29 eV, respectively (Table 2). The emission peaks’ intensity decreased with the increase in concentration (Table 2), suggesting a quenching process in the mixed layer sustained by the aggregation mechanism [62,63]. Electrons are attracted by MNDI because of its strong acceptor nature, and the number of charges that can relax from the excited state to the ground state is reduced, leading to a lower emission intensity. A morphology characterized by aggregates that were relatively homogeneously spread in the layer, with their dimensions and density depending on the concentration, was revealed in the SEM images (Figure 6g–i).

The emission peaks’ intensity increased with increasing concentration for the G1PPT and MNDI layers and decreased with increasing concentration for the G1PPT:MNDI mixed layers.

The energy of the emission peaks was lower than the band gap energy because the photoluminescence spectra were the result of competitive radiative and non-radiative relaxation processes, such as electron–hole scattering, electron–hole separation, and electron–hole emission. Among other factors, the photoluminescence could be affected by the presence of shallow and deep defects and energetic states introduced into the band gap by impurities and structural defects. 

On the G1PPT diffractogram, we did not identify any diffraction peaks (Figure 4a), confirming the disorder. The diffraction peaks revealed on the diffractogram of the MNDI layers indicated a certain degree of order in the layers, which was associated with a certain degree of crystallization (Figure 4b). This behavior suggests the presence of crystalline aggregates in the MNDI layer, with the sharp, narrow peaks being correlated with the presence of large crystalline zones. On the X-ray diffractograms of the samples with G1PPT:MNDI mixed layers, we identified peaks situated at the same positions as MNDI (Figure 4c). This result indicates that some degree of order was induced, most probably by the crystallization of MNDI. The X-ray diffractograms were similar for the MNDI and G1PPT:MNDI layers. 

The lack of specific diffraction peaks in the G1PPT layers confirmed the disorder, with the only ordering in the mixed layer being induced by MNDI. 

The surface of the G1PPT layer was smooth at concentrations of 0.025 g/2 mL and 0.1 g/2 mL (Figure 5a,c) and rougher at a concentration of 0.05 g/2 mL (Figure 5b). The MNDI layer was characterized by a morphology showing mountains and valleys, delimiting bulky aggregates of grains with narrow peaks (Figure 5d). These aggregates became larger, showing extended, elongated plateaus with the increase in concentration (Figure 5e,f). The roughness of the layer’s surface increased when the concentration increased from 0.025 to 0.05 and 0.1 g/2 mL. The presence of aggregates was also confirmed by the optical images (Figure 7d–f). The aggregates could be correlated with the ordered zones, which were revealed by the XRD analysis, especially for MNDI concentrations of 0.05 g/mL and 0.1 g/mL (Figure 7e,f). The mixed layers prepared from solutions with concentrations of 0.025 g/2 mL and 0.05 g/2 mL (Figure 5g,h) showed a morphology characterized by large aggregates/clusters, which was also confirmed by the optical images (Figure 7g,h). The roughness of the mixed layer decreased with the increase in concentration from 0.025 g/2 mL to 0.1 g/2 mL (Table 3). In the mixed layers, both clusters’ dimensions decreased with the increasing concentration, which was confirmed by the optical images (Figure 7g–i). The mountain–valley height difference also decreased with increasing concentration. Thus, a smoother surface on the mixed film was generated upon increasing the concentration. The tight interpenetration of the two phases represented by the dendrimer and naphthalene diimide and a significantly more homogeneous morphology—as also identified in the optical images (Figure 7i)—were obtained in the mixed layers of G1PPT:MNDI with the highest concentration. 

The mixed layer was smooth and uniform and covered the surface of the substrate, with no cracks or large grains/clusters.

The morphological and structural properties of the layers affect the electrical behavior of the heterostructures manufactured with these layers of ITO and Au electrodes. The interfaces of the organic layer with these electrodes could introduce important limitations, particularly by hindering charge carrier transport. The increase in the dendrimer concentration from 0.025 g/2 mL to 0.05 g/2 mL and 0.1 g/2 mL did not lead to an increase in the current passing through the heterostructure at the same applied bias. Charge carrier transport across the layer is not favored by the closer arrangement of the dendrimer units, as the dendrimers are rigid with little geometrical flexibility [64,65]. Neither Schottky emission (ln(I)~V^1/2^) nor Fowler–Nordheim tunneling (ln(I/V^2^)~1/V) is applicable regarding the conduction mechanism in the heterostructure with a single dendrimer layer, i.e., ITO/G1PPT/Au. However, the linear increase in the current with the voltage at low applied voltages (Figure 8a) suggests a dominant mechanism consisting of charge injection through direct tunneling [66]. For a high concentration (0.1 g/mL), at a forward bias, the linear fit of the log(I)–log(V) curve corresponds to conventional ohmic behavior (Figure 8b). At a reverse bias, the slight sub-ohmic behavior revealed for very low voltages becomes ohmic at voltages > 0.25 V (Figure 8b). The charge transport mechanism is not affected by the space-charge limitation (SCL) or trap-charge limitation (TCL), as confirmed by the slope being close to 1 (*p*~1) for the log(I)–log(V) dependence at both forward and reverse biases (Figure 8b).

In essence, charge carrier transport from one planar molecule of MNDI to another molecule of MNDI in the ITO/MNDI/Au heterostructure is favored in the layers prepared from a high-concentration solution. However, the measurements revealed that the current was lower when a higher-concentration solution was used to prepare the MNDI layer (Figure 9) because, at a higher concentration, the generation of aggregates is favored and the regular molecular orientation necessary for good charge carrier transport is disturbed [67], and this favors the generation of aggregates. At forward polarization, the log(I)–log(V) representation is linear and the slope changes from *p* < 1, corresponding to slightly sub-ohmic behavior for voltages < 0.45 V, to *p* >1 related to slightly supra-ohmic behavior for voltages > 0.45 V (Figure 9b). At reverse polarization, the slope changes slightly, corresponding to an increase in supra-ohmic behavior for voltages higher than 0.3 V (Figure 9b). At a higher concentration (0.1 g/2 mL), we observed injection contact behavior, showing significant asymmetry at reverse polarization (Figure 9a). For a given type of polarization, the current is lower than in the case of the heterostructure with an active layer prepared from the low-concentration solution, i.e., 0.025 g/2 mL (Figure 9a). For the layers prepared from the higher-concentration solution, i.e., 0.1 g/mL, under a forward bias, the slope of the linear log(I)–log(V) representation is *p*~1, corresponding to ohmic behavior (Figure 9c). Under a reverse bias (Figure 9c), there are two regions with slightly different coefficients of the power law. Region 1 shows a slope of *p*~1, indicating ohmic behavior for voltages < 0.25 V. Region 2 is characterized by a slope of *p* = 1.2, which suggests a slight deviation from linearity and the appearance of super-ohmic behavior for voltages > 0.25 V.

The interactions between the gold atoms (work function of gold = 5.2 eV [68]) and the organic molecules can determine the appearance of permanent dipoles at the gold–G1PPT and gold–MNDI interfaces, affecting the height of the contact barrier and the tunneling length of the charges across the interface [68]. These dipoles are the result of the interaction between the metal atoms and the organic molecules, involving different interactions, such as the polarization of the electrons of the organic molecules, partial charge transfer via covalent bonds between the metal and organic, the tunneling of the charge across the metal–organic interface, and the diffusion of the metal atoms [68,69,70]. Additionally, the organic layer surface or the region near the surface can be damaged during the deposition of Au contact. At the interface, the separation of the charge between the metal and organic occurs, generating interfacial dipoles and interfacial states on the organic side [68]. The injection of electrons from Au to G1PPT is unlikely because of the difference between the LUMO of G1PPT, situated at 2.99 eV, and the work function of gold, which is 5.2 eV. Electron injection is more likely to occur from Au to MNDI because of the lower energetic barrier between the LUMO of MNDI and the work function of gold, with the current being higher for heterostructures with an MNDI layer compared to those with a G1PPT layer. These dipoles can decrease/increase the energetic barrier for hole transfer from Au to the organic and from the organic to Au [68]. The hole injection at ITO/G1PPT and ITO/MNDI is similar because the barrier heights, which are determined by the ITO’s work function and the HOMO of the organic (6.45 eV for G1PPT and 6.36 eV for MNDI), are practically equal. Thus, the current in the heterostructures realized with the G1PPT dendrimer is comparable to the current in the same heterostructure realized with an MNDI layer, as the LUMO level of MNDI is lower on the energy scale than that of G1PPT. We expected a high current in the heterostructure with the MNDI layer because of the lower contact barrier at the MNDI/Au interface compared to the G1PPT/Au interface. However, the morphology of the organic layer may also play an important role in the transport of the charge carriers, as the boundaries of the aggregates are involved in the scattering and/or recombination of the charge carriers. Additionally, the roughness of the organic layer affects the quality of the MNDI/Au contact and, as a consequence, the efficiency of the charge carrier collection by the electrode. A large grain morphology favors better contact with the electrode. 

**Figure 10:**
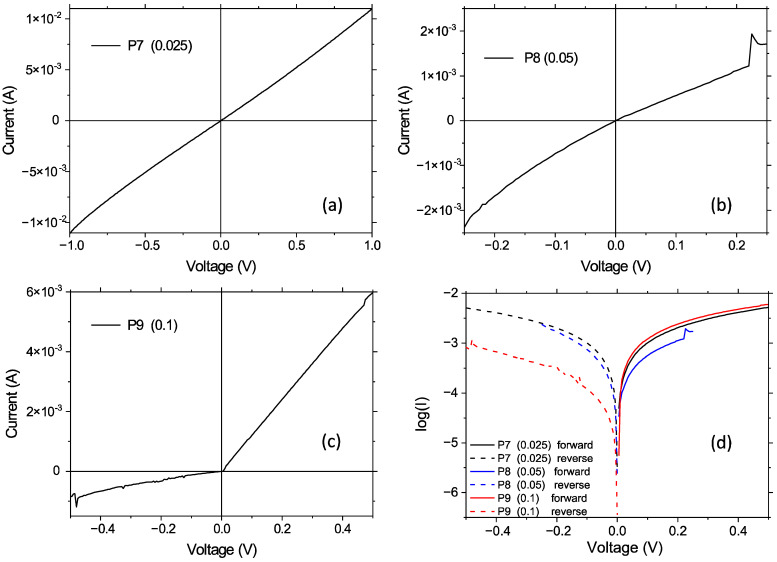
I–V (**a**–**c**) and log(I)–V (**d**) representations at forward and reverse polarization for glass/ITO/G1PPT:MNDI/Au heterostructures with mixed layers of different concentrations: 0.025 g/2 mL:0.025 g/2 mL (P7); 0.05 g/2 mL:0.05 g/2 mL (P8); 0.1 g/2 mL:0.1 g/2 mL (P9). The codes of the heterostructures are presented in Table 4.

The linear I = f(V) representation of the heterostructures with mixed layers, i.e., ITO/G1PPT:MNDI/Au, showed a change from linear to non-linear asymmetric behavior with the increase in concentration from 0.025 g/2 mL to 0.1 g/2 mL (Figure 10a–c). The semi-logarithmic I–V curve at forward and reverse biases for all heterostructures with the G1PPT:MNDI mixed layer showed a very small linear region at very low voltages, namely, around 0 V, and a significant deviation from linearity at voltages higher than 0.05 V (Figure 10d).

Currents of around 10^−2^–10^−3^ A were measured in the heterostructures with the G1PPT:MNDI mixed layer. This conduction was good compared to the conduction exhibited by the previously investigated heterostructures based on a new mixed layer with different donor–acceptor weight ratios, deposited via matrix-assisted pulsed laser evaporation (MAPLE) or vacuum evaporation. These included a mixed layer of an arylene-based polymer [poly[N-(2 ethylhexyl)2.7-carbazolyl vinylene and poly[N-(2-ethylhexyl)2.7-carbazolyl 1.4-phenylene ethynylene]]–C60, with ~10^−9^–10^−10^ A at 0.5 V [71]; arylenevinylene-based oligomers [1,4-bis [4-(N,N diphenylamino)phenylvinyl] benzene and 3,3-bis(N-hexylcarbazole)vinylbenzene]–C60, with ~10^−10^ A at 0.5 V [72]; and an oligoazomethine [N,N-bis[(N-hexyl 3-carbazolyl)benzylidene] 2,5-diamino-3,4-dicyanothiophene and N,N-bis[(4 diphenylamino)benzylidene] 2,5-diamino-3,4-dicyanothiophene]–fullerene derivative mixed layer, with ~10^−8^ A at very low voltages [73]. However, the obtained conduction was comparable to that of heterostructures with star-shaped arylenevinylene compounds [4,4′,4″-tris[(4′-diphenylamino) styryl] triphenylamine]–[6,6]-phenyl C61, a butyric acid butyl ester, with ~10^−4^ A at 0.5 V [74], and heterostructures with a mixed layer of a star-shaped arylenevinylene compound and non-fullerene compound [N,N′-bis-(1-dodecyl)perylene-3,4,9,10 tetracarboxylic diimide], with ~10^−3^ A at 0.3 V [75]. The current density in the heterostructures composed of the dendrimer/naphthalene diimide derivative was approximately 10^−3^ A/cm^2^ at 0.5 V, higher than the current density of heterostructures realized with the most used donor and acceptor, zinc phthalocyanine (ZnPc) and (10,15,20-tetra(4-pyridyl) 21H,23H-porphyne (TPyP), which were < 10^−8^ A/cm^2^ at 0.5 V [76].

**Figure 11:**
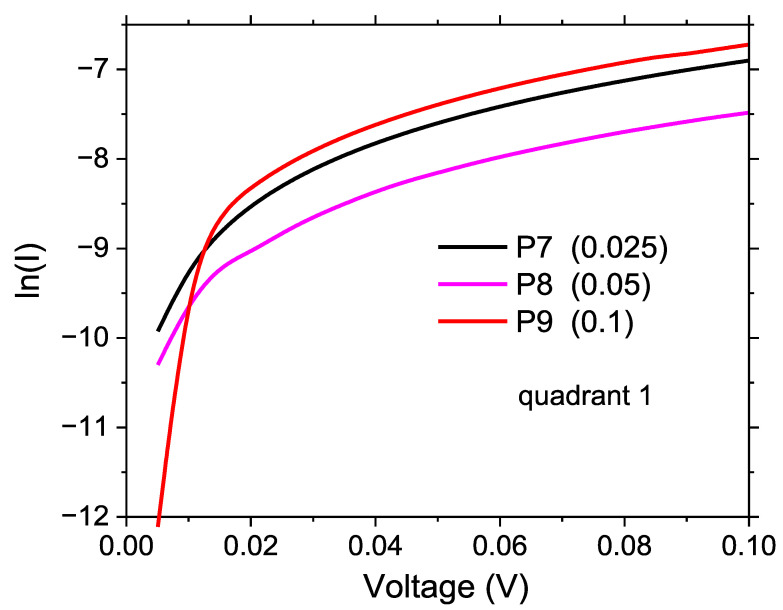
ln(I)–V representation at forward polarization (ITO−, Au+) for the glass/ITO/G1PPT:MNDI/Au heterostructures with mixed layers of different concentrations: 0.025 g/2 mL:0.025 g/2 mL (P7); 0.05 g/2 mL:0.05 g/2 mL (P8); 0.1 g/2 mL:0.1 g/2 mL (P9).

On the ln(I) = f(V) curves of samples P7, P8, and P9 (Figure 11), we identified voltage ranges exhibiting different slopes and, as a consequence, different ideality factors for the ITO/organic mixed layer/Au Schottky diodes. Thus, for a low applied voltage (<0.1 V), we obtained an ideality factor of < 3 for all samples (Table 4) with mixed layers, namely, P7, P8, and P9, for voltages < 0.1 V. The ideality factors of the organic Schottky diodes presented in the literature vary from 2 to 11 [77]. Here, the ideality factor increased with the increase in applied voltage, reaching a value higher than 6 (reaching values of tens) at voltages higher than 0.25 V.

Independent of the concentration, the values of the ideality factor indicate non-ideal behavior, which could be due to the recombination of the charge carriers favored by the generation of the interfacial layer and imperfections during the deposition of the layer.

The log I = f(V) representation revealed a variation in the ratio between the current at forward and reverse polarization at a fixed voltage (e.g., 0.25 V) for the heterostructure with mixed layers prepared from solutions with different concentrations (Figure 10d). This ratio is correlated with rectifying behavior, i.e., reduced charge carrier injection through the organic layer at reverse polarization compared to the charge carrier injection at forward polarization [78,79]. The curve for the heterostructures with mixed layers prepared from low-concentration solutions (0.025 g/2 mL and 0.05 g/2 mL) did not show significant differences in the behavior at forward and reverse polarization. Therefore, these heterostructures do not behave as rectifier contacts. The rectification ratio (η) of the heterostructure with an active layer of a higher concentration (0.1 g/2 mL) and a smooth surface (Table 3) was approximately eight times higher than the η of the heterostructure with mixed layers of low concentrations (0.05 g/2 mL and 0.025 g/2 mL) and a rough surface (Table 3). The rectifying behavior is affected by both the morphology of the organic layer and the height of the contact barrier between the organic layer and electrodes [80]. The morphology determines the interface of the organic electrode with an effect on charge carrier collection. The height of the contact barrier between the organic layer and electrodes determines the number of charge carriers surpassing the barrier. The rectification is more significant at high concentrations because the layer is smooth, enabling good contact with the gold electrode. Additionally, the barriers that must be surpassed by electrons at direct polarization (ITO−; Au+) are lower than the barriers for holes at reverse polarization (ITO+, Au−). 

The concentration of the mixed layer based on the propylene thiophenoimine dendrimer donor and naphthalene diimide derivative acceptor affects the balance between the forward and reverse currents passing through the ITO/mixed layer/Au heterostructure, and thus, the rectifier behavior. As a consequence, changing the concentration of the mixed layer could offer a means to obtain rectifier properties for heterostructures based on these mixed layers, which is promising for a large number of electronic applications.

## 5. Materials and Methods

**Figure 12:**
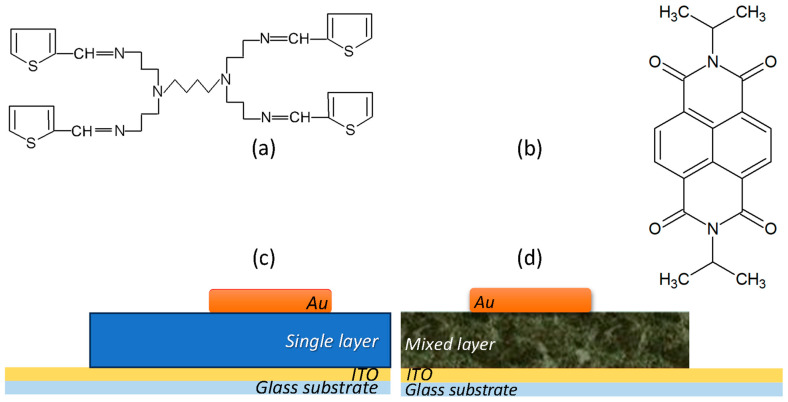
Chemical structures of (**a**) generation 1 poly(propylene thiophenoimine) (G1PPT) and (**b**) N,N′-diisopropylnaphthalene diimide/MNDI; (**c**) organic heterostructure with a single layer, G1PPT or MNDI prepared via spin coating; (**d**) organic heterostructure with mixed-layer G1PPT:MNDI prepared via spin coating.

The donor compound, the generation 1 poly(propylene thiophenoimine) (G1PPT) dendrimer (Figure 12a), was obtained via the functionalization of a generation 1 poly(propylene imine) tetraamine with thiophene in a condensation reaction using 2-thiophene carbaldehyde, followed by stirring the mixture under a nitrogen atmosphere. G1PPT was collected after the solvent’s evaporation and the washing of the resulting product in a mixture of dichloromethane and water. To prepare the acceptor, N,N′- diisopropylnaphthalene diimide (Figure 12b), 1, 4, 5, 8-naphthalene tetracarboxylic dianhydride, N,N-dimethylformamide, and isopropylamine were mixed and stirred at 110 °C. The obtained material was filtered, immersed in ice-cold water, and filtered again to obtain MNDI. Details of the synthetic routes have been presented previously: Refs. [46,47,81] for G1PPT and Refs. [46,47,81,82,83] for MNDI. 

To avoid significant impurification of the synthesized compounds, the reagents used in the synthesis of the compounds had purity > 99%, and the reaction vessel was subjected to a specific cleaning procedure, including washing, rinsing, and drying, to remove the moisture, followed by cleaning with nitrogen gas [30,46]. The prepared G1PPT and MNDI were analyzed via Fourier-transform infrared spectroscopy, and the absorption peaks characteristic of the functional groups specific to these compounds were revealed: 1632 cm^−1^, attributed to C=N bonds in the dendrimer moiety, and 789 cm^−1^, corresponding to the out-of-plane vibration of the thiophene ring [46] in G1PPT, as well as 2830 and 2938 cm^−1^, corresponding to C-H stretching (isopropyl group) in MNDI [47]. 

Single layers of G1PPT and MNDI were prepared on different substrates with dimensions of 25 mm × 12 mm × 1.1 mm (quartz glass; glass covered with indium tin oxide: glass/ITO—transmission > 85%, sheet resistance <15 Ω/□; thickness = 155 nm; single-crystal silicon: Si). Deposition was performed from a solution via spin coating using Brewer Science equipment in a one-step deposition process.

The substrates were cleaned ultrasonically in water with a detergent for 5 min followed by acetone for 5 min and ethyl alcohol for 5 min; after this, they were dried via purging with dry nitrogen. Oxygen plasma treatment for 5 min rendered the deposition substrates hydrophilic for a short period of time, namely, around 1 h, favoring the deposition of the organic layers.

Chloroform (Ch) was selected as a solvent, considering the solubility of the dendrimer and naphthalene diimide derivative at room temperature. “Mother solutions” with three different concentrations were prepared by dissolving the same quantity of each component, G1PPT and MNDI, at 0.025 g, 0.05 g, or 0.1 g, in 2 mL of chloroform. The layers were deposited with a rotation speed of 4000 rpm for a duration of 30 s.

The mixed active layers (Figure 1d) were prepared on the same substrates by dissolving the same quantity (0.025 g, 0.05 g, or 0.1 g) of G1PPT and MNDI in 2 mL of solvent and then mixing these two solutions to prepare three “mother solutions” for the mixed layers. The deposition conditions for the mixed layers were the same as for the single layers: rotation speed = 4000 rpm and duration = 30 s.

An Ambios Technology XP100 profilometer was used to measure the thickness of the active layer considering the step between the ITO layer and organic layer. We considered the average values of three measurements performed at different points on each sample. Thus, depending on the concentration, we obtained a value between 24 nm and 75 nm for the G1PPT layer, between 59 nm and 80 nm for the MNDI layer, and between 25 nm and 40 nm for the G1PPT:MNDI mixed layer.

On top of the single (Figure 12c) and mixed layers (Figure 12d) deposited on glass/ITO, the second electrode was deposited via sputtering (deposition duration = 6 min). This consisted of a layer of gold (Au) with an area of 0.28 cm^2^ and a thickness of 50 nm. 

UV–Vis transmission spectroscopy, performed with a Carry 5000 dual-beam spectrophotometer, was used to investigate the optical properties of the single and mixed in the spectral range of 250–800 nm. To highlight all details of the transmission spectra, the layers were deposited on quartz substrates.

The emission properties of the deposited single layers, stacked bi-layers, and mixed layers were revealed by the photoluminescence (PL) spectra, which were obtained using an Edinburgh Instruments F-900 Spectrofluorimeter at a UV excitation wavelength of λ_excitation_ = 335 nm (Δλ = 350–650 nm), with a slit of 0.5 or 1.5 for samples showing strong signals. The photoluminescence emission properties were investigated on layers deposited on Si substrates because Si does not show emission at the excitation wavelength of 335 nm.

The structural particularities of the layers were analyzed via X-ray diffraction (XRD) measurements, which were realized with a Bruker D8 Advance Diffractometer, working in locked-coupled mode, using the K_α_ line of Cu and the following experimental parameters: acceleration voltage = 40 kV, anode current = 40 mA, angular range = 5–60°, increment = 0.02°, scan speed = 0.1 s/step, and slit = 0.6.

Atomic force microscopy (AFM) provided details about the topography of the film surface for the single layers and mixed layers. We used a MultiView 4000 Nanonics System working in tapping mode: scan area = 40 µm × 40 µm, scan speed = 6.12 lines/s, scan resolution = 256 lines, probe diameter = 20 nm, vibration frequency = 37 kHz, and factor of merit = 1900. We obtained information about the roughness of the organic films, evaluating the root mean square (RMS) and roughness average (RA) with Gwyddion software version 2.59. The RMS and RA values were the average values of at least two measurements realized on each sample; for a suitable comparison between the samples, we scanned the same area. Because some layers showed large variations in thickness, the roughness could be larger than the layer’s average thickness.

Information about the morphology of the layers deposited on the Si substrate was obtained via scanning electron microscopy (SEM), which was performed with a Zeiss EVO 50 XVP microscope in top view (V_acc_ = 4 kV, 10 kV; working distance = 8 mm; magnification = 100×; 200×). The morphology was investigated using the optical images of the deposited films, obtained with an Olympus microscope, which was part of the Nanonics 4000 MultiView configuration, at a magnification of 100×.

The electrical properties of the heterostructures with single layers and mixed layers, namely, glass/ITO/G1PPT/Au, glass/ITO/MNDI/Au, and glass/ITO/G1PPT:MNDI/Au, were evaluated by drawing the I–V characteristic curves in the dark in a vertical configuration using a SouceMeter Keithley 2400 [72,84,85] and a transversal measurement geometry with three wires: one wire in direct contact with the Au electrode and two wires in direct contact with the ITO electrode. All measurements were carried out under ambient temperature and pressure conditions.

## 6. Conclusions

This work studied the properties of organic heterostructures containing a dendrimer, poly(propylene thiophenoimine) (G1PPT), a naphthalene diimide derivative, N,N′-diisopropylnaphthalene diimide (MNDI) single layer, and G1PPT:MNDI mixed layers, prepared from a solution via spin coating. The optical and electrical properties of the heterostructures with single G1PPT and MNDI layers were explored through a comparison with the properties of heterostructures with a mixed G1PPT:MNDI layer.

The band gap of the G1PPT layer increased with increasing concentration and the band gap of the G1PPT:MNDI layer slightly decreased with increasing concentration. The band gap of the mixed layer was wider compared to the band gap of the individual components, G1PPT and MNDI, because mixing generated a certain degree of order in the layer, limiting the introduction of the trap levels. 

An absorption mechanism involving direct allowed transitions was identified in the G1PPT and MNDI layers, with the layer of MNDI showing a lower degree of disorder than the G1PPT layer at low concentrations. At high concentrations, the degree of disorder in the MNDI layer was higher than that in the G1PPT layer as a consequence of the presence of aggregates, which were developed in the MNDI layer during deposition. The lowest Urbach energy value, correlated with a reduced degree of disorder, was obtained at a concentration of 0.1 g/2 mL for the G1PPT and G1PPT:MNDI layers. For the MNDI layers, we obtained the highest E_u_ at a concentration of 0.1 g/2 mL.

The emission peaks’ intensity increased with concentration for the G1PPT and MNDI layers because the number of excited units that could relax to the ground state increased. The shape and intensity of the emission of MNDI were probably due to the excimer-like emission that was present in the solid state. The emission peaks’ intensity in the mixed layer decreased with increasing concentration, suggesting a quenching process sustained by the aggregation mechanism. 

The dendrimer layers obtained via spin coating were smoother than the layers of the naphthalene diimide derivative deposited via spin coating. The MNDI layers became rougher with the increase in concentration, while the roughness of the mixed layers decreased with the increase in concentration. These morphological particularities are very important for the type of contact with the gold electrode, which, in turn, affect the electrical properties.

Most of the heterostructures showed ohmic behavior, and for a given voltage, the current increased with the concentration. An exception was the ITO/MNDI/Au structure because the molecular orientation was perturbed at high concentrations, and this favored the generation of aggregates, leading to charge carrier loss through scattering or recombination at the boundaries. The behavior of the heterostructures with mixed layers changed from linear to non-linear asymmetric with the increasing concentration of the layer, and its rectifier behavior became significant. 

Independent of the concentration, the values of the ideality factor for the heterostructures with mixed layers indicated non-ideal behavior, which could have been due to the recombination of the charge carriers, favored by the generation of the interfacial layer and imperfections during the deposition of the layer.

These organic heterostructures with layers prepared from a poly(propylene thiophenoimine) dendrimer, a N,N′-diisopropylnaphthalene diimide, and their mixture showed good conductivity (currents on the order of mA) and are promising for application as conductive layers in organic electronic devices. Rectifier behavior was revealed for the heterostructure based on a mixed layer of dendrimer/naphthalene diimide derivative with a concentration of 0.1 g/2 mL, and this feature could be useful for many electronic applications. Thus, further developments may consider the effect of the mixed-layer composition on the rectification property.

## Figures and Tables

**Table 1:** Band gaps and Urbach energies for the single layers and mixed layers deposited on quartz glass substrates.

Code	Structure	E_g_ (eV)	E_u_ (eV)
Q1	Quartz/G1PPT (0.025 g/2 mL)	3.41 ± 0.04	5.10 ± 0.05
Q2	Quartz/G1PPT (0.05 g/2 mL)	3.44 ± 0.04	2.82 ± 0.06
Q3	Quartz/G1PPT (0.1 g/2 mL)	4.00 ± 0.04	0.621 ± 0.003
Q4	Quartz/MNDI (0.025 g/2 mL)	3.03 ± 0.06	0.715 ± 0.005
Q5	Quartz/MNDI (0.05 g/2 mL)	3.06 ± 0.04	0.520 ± 0.004
Q6	Quartz/MNDI (0.1 g/2 mL)	2.93 ± 0.04	0.718 ± 0.005
Q7	Quartz/G1PPT (0.025 g/2 mL):MNDI (0.025 g/2 mL)	4.53 ± 0.03	0.494 ± 0.003
Q8	Quartz/G1PPT (0.05 g/2 mL):MNDI (0.05 g/2 mL)	4.50 ± 0.04	0.602 ± 0.005
Q9	Quartz/G1PPT (0.1 g/2 mL):MNDI (0.1 g/2 mL)	4.38 ± 0.07	0.375 ± 0.002

**Table 2:** Deconvolution parameters of PL spectra at excitation with 335 nm for layers of different concentrations: G1PPT [S1—0.025 g/2 mL; S2—0.05 g/2 mL]; MNDI [S3—0.1 g/2 mL; S4—0.025 g/2 mL; S5—0.05 g/2 mL; S6—0.1 g/2 mL]; G1PPT:MNDI [S7—0.025 g/2 mL:0.025 g/2 mL; S8—0.05 g/2 mL:0.05 g/2 mL; S9—0.1 g/2 mL:0.1 g/2 mL].

Code	Structure	E (eV)	FWHM (eV)	Peak Intensity (Count)
S1	G1PPT (0.025)	2.41; 2.57; 2.88	0.47; 0.25; 0.43	2.26 × 10^4^; 1.059 × 10^4^; 0.991 × 10^4^
S2	G1PPT (0.05)	2.40; 2.57; 2.89	0.50; 0.25; 0.39	3.42 × 10^4^; 1.115 × 10^4^; 0.864 × 10^4^
S3	G1PPT (0.1)	2.39; 2.56; 2.86	0.47; 0.25; 0.45	8.36 × 10^4^; 2.72 × 10^4^; 1.799 × 10^4^
S4	MNDI (0.025)	2.42; 2.58	0.44; 0.26	2.09 × 10^5^; 2.59 × 10^5^
S5	MNDI (0.05)	2.44; 2.58	0.44; 0.27	3.82 × 10^5^; 5.59 × 10^5^
S6	MNDI (0.1)	2.46; 2.59	0.40; 0.26	8.87 × 10^5^; 12.71 × 10^5^
S7	G1PPT:MNDI (0.025)	2.30; 2.37; 2.57	0.33; 0.21; 0.29	2.71 × 10^5^; 3.12 × 10^5^; 22.2 × 10^5^
S8	G1PPT:MNDI (0.05)	2.30; 2.37; 2.57	0.35; 0.21; 0.29	2.07 × 10^5^; 2.22 × 10^5^; 15.33 × 10^5^
S9	G1PPT:MNDI (0.1)	2.29; 2.37; 2.58	0.32; 0.21; 0.29	1.158 × 10^5^; 1.461 × 10^5^; × 10.71 × 10^5^

**Table 3:** Roughness parameters for single (G1PPT, MNDI) and mixed (G1PPT:MNDI) layers with different concentrations (0.025 g/2 mL; 0.05 g/2 mL; 0.1 g/2 mL) deposited on glass/ITO.

Code	Sample	RMS (nm)	RA (nm)
P1	Glass/ITO/G1PPT (0.025 g/2 mL)	3.77	1.83
P2	Glass/ITO/G1PPT (0.05 g/2 mL)	37.4	23.1
P3	Glass/ITO/G1PPT (0.1 g/2 mL)	4.89	2.16
P4	Glass/ITO/MNDI (0.025 g/2 mL)	69.7	52.0
P5	Glass/ITO/MNDI (0.05 g/2 mL)	132.4	92.8
P6	Glass/ITO/MNDI (0.1 g/2 mL)	157.5	108.0
P7	Glass/ITO/G1PPT (0.025 g/2 mL):MNDI (0.025 g/2 mL)	265	201
P8	Glass/ITO/G1PPT (0.05 g/2 mL):MNDI (0.05 g/2 mL)	110.9	70.3
P9	Glass/ITO/G1PPT (0.1 g/2 mL):MNDI (0.1 g/2 mL)	11.76	9.33

**Table 4:** Ideality factors of diodes with mixed G1PPT:MNDI layers at different concentrations, i.e., 0.025 g/2 mL:0.025 g/2 mL (P7); 0.05 g/2 mL:0.05 g/2 mL (P8); and 0.1 g/2 mL:0.1 g/2 mL (P9), for voltages <0.1 V.

Code	Heterostructure	Voltage (V)	Ideality Factor (n)
P7	Glass/ITO/G1PPT:MNDI (0.025)/Au	<0.025	n < 1
		0.025–0.05	1 < n < 2
		0.05–0.1	2 < n < 3
P8	Glass/ITO/G1PPT:MNDI (0.05)/Au	≤0.025	n < 1
		0.025–0.05	1 < n < 1.5
		0.05–0.1	1.5 < n < 3
P9	Glass/ITO/G1PPT:MNDI (0.1)/Au	≤0.025	n < 1
		0.025–0.05	1 < n < 1.5
		0.05–0.1	1.5 < n < 3

## Data Availability

The dataset is available upon request from the authors.
